# Macrophage migration inhibitory factor confers resistance to senescence through CD74-dependent AMPK-FOXO3a signaling in mesenchymal stem cells

**DOI:** 10.1186/s13287-015-0076-3

**Published:** 2015-04-22

**Authors:** Wenzheng Xia, Fengyun Zhang, Congying Xie, Miaomiao Jiang, Meng Hou

**Affiliations:** Department of Neurosurgery, First Affiliated Hospital, Wenzhou Medical University, Wenzhou, 325000 PR China; Key Laboratories of Education Ministry for Myocardial Ischemia Mechanism and Treatment, The 2nd Affiliated Hospital of Harbin Medical University, Harbin, 150086 PR China; Department of Cardiology, The 2nd Affiliated Hospital of Harbin Medical University, Harbin, 150086 PR China; Department of Radiation Oncology, First Affiliated Hospital, Wenzhou Medical University, Wenzhou, 325000 PR China

## Abstract

**Introduction:**

Mesenchymal stem cells (MSCs)-based therapies have had positive outcomes in animal models of cardiovascular diseases. However, the number and function of MSCs decline with age, reducing their ability to contribute to endogenous injury repair. The potential of stem cells to restore damaged tissue in older individuals can be improved by specific pretreatment aimed at delaying senescence and improving their regenerative properties. Macrophage migration inhibitory factor (MIF) is a proinflammatory cytokine that modulates age-related signaling pathways, and hence is a good candidate for rejuvenative function.

**Methods:**

Bone marrow-derived mesenchymal stem cells (BM-MSCs) were isolated from young (6-month-old) or aged (24-month-old) male donor rats. Cell proliferation was measured using the CCK8 cell proliferation assay; secretion of VEGF, bFGF, HGF, and IGF was assessed by RT-qPCR and ELISA. Apoptosis was induced by hypoxia and serum deprivation (hypoxia/SD) for up to 6 hr, and examined by flow cytometry. Expression levels of AMP-activated protein kinase (AMPK) and forkhead box class O 3a (FOXO3a) were detected by Western blotting. CD74 expression was assayed using RT-qPCR, Western blotting, and immunofluorescence.

**Results:**

In this study, we found that MSCs isolated from the bone marrow of aged rats displayed reduced proliferative capacity, impaired ability to mediate paracrine signaling, and lower resistance to hypoxia/serum deprivation-induced apoptosis, when compared to younger MSCs. Interestingly, pretreatment of aged MSCs with MIF enhanced their growth, paracrine function and survival. We detected enhanced secretion of VEGF, bFGF, HGF, and IGF from MIF-treated MSCs using ELISA. Finally, we show that hypoxia/serum deprivation-induced apoptosis is inhibited in aged MSCs following MIF exposure. Next, we found that the mechanism underlying the rejuvenating function of MIF involves increased CD74-dependent phosphorylation of AMPK and FOXO3a. Furthermore, this effect was abolished when CD74, AMPK, or FOXO3a expression was silenced using small-interfering RNAs(siRNA).

**Conclusions:**

MIF can rejuvenate MSCs from a state of age-induced senescence by interacting with CD74 and subsequently activating AMPK-FOXO3a signaling pathways. Pretreatment of MSCs with MIF may have important therapeutic implications in restoration or rejuvenation of endogenous bone marrow-MSCs in aged individuals.

## Introduction

Despite significant advances in the medical management of heart failure, ischemia/reperfusion injury continues to be a leading cause of death in developed countries [1]. In the last few years, many investigators have shown that transplantation of bone marrow-derived mesenchymal stem cells (MSCs) is a promising tool for the repair and regeneration of cardiomyocytes as well as restoration of heart function [2-4]. However, clinical studies together with animal studies have shown that the regenerative potential of bone and other tissues declines progressively with age [5]. Consequently, transplantation of MSCs derived from older donors appears to be less effective than their younger counterparts [6]. If the age-dependent decrease in regenerative potential is caused by intrinsic changes in MSCs themselves, autologous cell therapy approaches are prone to be suboptimal in older patients, who incidentally are in most need of such treatment procedures [7]. Therefore, an optimum therapeutic strategy for diseases associated with old age could be identification of ways to replenish stem cell function by, for instance, rejuvenating endogenous stem/progenitor cells that might infiltrate and supply the ischemic tissue with new blood vessels to prevent tissue damage [8-10].

Macrophage migration inhibitory factor (MIF) is a pleiotropic cytokine that maintains homeostasis at set-point levels by regulating physiological signaling pathways [11]. MIF is expressed in several cell types, including monocytes and macrophages, vascular smooth muscle cells and cardiomyocytes [12-14]. There is also evidence that MIF exerts a fundamental role in the metabolic response to environmental stress [11,15]. In the heart, MIF is released by ischemic cardiomyocytes, leading to enhanced glucose uptake and protection from ischemic injury and cellular apoptosis. In addition, studies have shown that MIF expression is regulated by senescence, and that in aged hearts its secretion is significantly reduced. This in turn leads to dysregulation of glucose uptake during both ischemia and reperfusion, which is likely to account for reduced tolerance to ischemic stress in older individuals. However, researchers have also shown that regaining MIF function attenuated such an injury [16]. Furthermore, MIF also contributes to cell survival and proliferation, and has been shown to prevent cellular senescence [15,17]. In this study, we investigate whether MIF could rejuvenate aged MSCs and enhance their function, so they could be applied to the treatment of ischemic heart diseases.

Previous research has shown that MIF acts through the AMP-activated protein kinase (AMPK) signaling pathway to induce cellular resistance to glucose deprivation, ischemia, hypoxia, oxidative and senescent stress [15,18]. Activation of AMPK can slow down the process of senescence [19], and has been investigated in mammals as a therapeutic target in age-related pathologies [20,21]. Interestingly, reduced AMPK activity in the aging heart can be recovered when treated with MIF. In the present study, we sought to examine whether AMPK activation by MIF could restore the survival and function of aged MSCs.

In addition to AMPK, we also explored the role of Forkhead box class O 3a (FOXO3a) as a possible modulator of MIF function during the rejuvenation of MSCs. FOXO3a is a downstream effector of AMPK signaling, and is known to be associated with human longevity [22]. In addition, it has been implicated in the regulation of diverse cellular functions including differentiation, metabolism, proliferation and cell survival [23,24]. Downregulation of FOXO3a expression can accelerate cellular senescence in human dermal fibroblasts and endothelial cells [25].

Based on the above evidence, we hypothesized that MIF activates AMPK–FOXO3a signaling to rejuvenate MSCs from cellular senescence. This study was designed to verify this hypothesis, and elucidate the influence of MIF on aged MSCs.

## Materials and methods

### Animals

Young and mature (6 months old) and old (24 months old) male Sprague–Dawley rats were maintained in accordance with guidelines published by the US National Institutes of Health. All study procedures were approved by the Harbin Medical University Institutional Animal Care and Use Committee. This study was conducted in compliance with the Guide for the Care and Use of Laboratory Animals published by the National Academy Press (National Institutes of Health, revised in 1996).

### Reagents

Dulbecco’s modified Eagle’s medium and fetal bovine serum were obtained from Hyclone (Logan, UT, USA). Trizol reagent was obtained from Invitrogen (Carlsbad, CA, USA). The First Stand cDNA Synthesis Kit, Fast Start Universal SYBR Master (ROX) and X-treme GENE HP DNA transfection reagent were obtained from Roche (Mannheim, Germany). The Annexin V–fluorescein isothiocyanate (FITC) Apoptosis Detection Kit and primary antibodies anti-CD44, anti-CD29 and anti-CD90 were obtained from BD Pharmingen (Franklin Lakes, NJ, USA). The primary antibodies anti-CD34 and anti-CD45 were obtained from eBioscience (San Diego, CA, USA). Rabbit monoclonal antibodies against AMPKα, phospho-AMPKα (Thr172), phospho-FoxO1(Thr24)/FoxO3a(Thr32) and FoxO3a were purchased from Cell Signaling Technology (Danvers, MA, USA). Rabbit monoclonal antibody anti-CD74 was obtained from Santa Cruz Biotechnology (Dallas, TX, USA). Mouse polyclonal antibody anti-β-actin was procured from Zhongshan Goldenbridge Biotechnology (#TA-09; Zhongshan Goldenbridge Biotechnology Co. Ltd, Zhongshan, Guang Dong, China). Horseradish peroxidase-conjugated anti-mouse and anti-rat secondary antibodies were obtained from Santa Cruz Biotechnology. Alexa Fluor 555-conjugated goat anti-rabbit IgG was procured from Invitrogen. MIF, vascular endothelial growth factor (VEGF), basic fibroblast growth factor (bFGF), hepatocyte growth factor (HGF) and insulin-like growth factor (IGF) enzyme-linked immunosorbent assay kits were obtained from Rapidbio (Winnetka, CA, USA). Small interfering RNAs (siRNAs) targeted to AMPK and FOXO3a transcripts were obtained from Life Technologies (Carlsbad, CA, USA). siRNA targeted to CD74 was obtained from QiaGen (Germantown, MD, USA). Rat recombinant MIF was obtained from Prospec (East Brunswick, NJ, USA). The cell proliferation assay, Cell Counting Kit-8 (CCK-8), was obtained from HaiGene Technology (Harbin, China).

### Cell culture and treatment

Bone marrow MSCs were isolated from the femur and tibia of Sprague–Dawley rats as described previously [26]. Briefly, bone marrow cells were flushed out from the femur and tibia using 5 ml Dulbecco’s modified Eagle’s medium/F12. Next, the red blood cells were lysed and removed, and the remaining cells (5 × 10^5^) were plated on a 25 cm^2^ flask in 6 ml Dulbecco’s modified Eagle’s medium/F12 supplemented with 10% fetal bovine serum and 1% penicillin/streptomycin. The cells were cultures at 37°C and 5% carbon dioxide. After 3 days in culture, the nonadherent cells were washed out, while the adherent MSCs were grown further in the above media, which was replaced every 3 days. Once the culture reached 80 to 90% confluency, the cells were trypsinized and passaged at 2:3 or 1:2 dilution. All cells used in subsequent assays belonged to passages 3 to 5.

The characteristic properties of MSCs were demonstrated by immunophenotyping. To verify the identity and biological relevance of cultured MSCs, cells were labeled using antibodies against various cell-surface markers and analyzed by flow cytometry. Briefly, cultured MSCs were harvested, washed with phosphate-buffered saline, and immunostained with the following antibodies: phycoerythrin-conjugated anti-CD45 and anti-CD90; and FITC-conjugated anti-CD44, anti-CD29 and anti-CD34. Labeled cells were assayed by flow cytometry, and analyzed using the FACSDiva Pro Software (Becton-Dickinson, San Jose, CA, USA).

For MIF stimulation, cells were fed with media containing 100 ng/ml recombinant MIF and incubated at 37°C for various durations of time as described previously [27].

To induce apoptosis *in vitro*, culture conditions were designed to mimic the hypoxia and serum deprivation (hypoxia/SD) associated with ischemic myocardium *in vivo*, in accordance with previous reports [28]. Briefly, MSCs were incubated in serum-free media in a controlled atmosphere (anaerobic chamber) glove box (Plas Labs 855-AC; Lansing, PLAS LABS, INC., MI, USA) to scavenge free oxygen. Cells exposed to hypoxia/SD alone were used as apoptotic controls. In the experimental condition, MIF (100 ng/ml) was added to the medium at the time of exposure to hypoxia/SD and the cultures were reincubated for 6 hours either in the absence or continued presence of MIF under hypoxic conditions.

### Cell proliferation assay

The rate of cell proliferation was estimated using the CCK-8 assay, which was performed according to the manufacturer’s protocol. Briefly, cells grown in a 96-well plate were incubated with the CCK-8 solutions for 1 hour at 37°C, following which the absorbance of each well at 450 nm was recorded.

### Enzyme-linked immunosorbent assay

The concentration of secreted MIF, VEGF, bFGF, HGF and IGF in the cell culture media was measured using an enzyme-linked immunosorbent assay kit. Assays were conducted in 96-well microplates according to the manufacturer’s instructions.

### Flow cytometric analysis of apoptosis

The extent of apoptotic cell death was assayed using the Annexin V–FITC Apoptosis Detection Kit, performed according to the manufacturer’s instructions, determined by detecting phosphatidylserine exposure on cell plasma membrane with the fluorescent dye. Briefly, cells were harvested and washed in ice-cold phosphate-buffered saline, resuspended in 300 μl binding buffer and incubated with 5 μl Annexin V–FITC solution for 30 minutes at 4°C in the dark. This was followed by incubation with 5 μl propidium iodide for 5 minutes. The samples were immediately analyzed by bivariate flow cytometry on the BD FACSCantoII equipped with Cell Quest software (BD Pharmingen, Becton-Dickinson, San Jose, CA). Approximately 1 × 10^5^ to 5 × 10^5^ cells were analyzed in each sample.

### Knockdown of gene expression using small interfering RNA

MSCs were transfected using the X-treme GENE HP DNA Transfection Reagent, according to the manufacturer’s instructions. Briefly, MSCs were cultured in a six-well plate treated with the transfection reagent in a 3:1 ratio of reagent to siRNA weight for 20 minutes, followed by addition of a mixture containing 100 nM siRNA, and were incubated in 2 ml culture medium for 48 hours. Scrambled small interfering RNA (siRNA-NT) was used as the control. Transfection efficiency of siRNA-CD74, siRNA-AMPK and siRNA-FOXO3a was determined by western blotting.

### Quantitative real-time PCR

The expression levels of several genes were analyzed by quantitative real-time PCR. Briefly, total cellular RNA was isolated and reverse transcribed using the transcriptor First Stand cDNA Synthesis Kit, according to the manufacturer’s instructions. The quantitative PCR was carried out using the Fast Start Universal SYBR Master and fluorescence quantitative PCR system [23]. The threshold number of cycles (Ct) was set within the exponential phase of the PCR reaction, and the △Ct value for each target gene was calculated by subtracting the Ct value of glyceraldehyde 3-phosphate dehydrogenase (internal control) from the target gene. Relative gene expression levels were calculated by comparing the △Ct values between control and experimental conditions for each target PCR, and calculated using the following equation:

Relative gene expression = 2^–(△Ct sample – △Ct control)^.

The primer pairs used to detect the mRNA levels of target genes are presented in Table 1.

**Table 1:** **Primer sequences**

**Gene**	**Sequences**
MIF	Forward: 5′-ATGAACTTTCTGCTGTCTTG-3′
	Reverse: 5′-TCACCGCCTCGGCTTGTCA-3′
VEGF	Forward: 5′-CAGCGACAAGGCAGACTATT-3′
	Reverse: 5′-GTTGGCACGATTTAAGAGGG-3′
bFGF	Forward: 5′-CAGCGACAAGGCAGACTATT-3′
	Reverse: 5′-CGTTTCAGTGCCACATACCA-3′
HGF	Forward: 5′-CGAGCTATCGCGGTAAAGAC-3′
	Reverse: 5′-TGTAGCTTTCACCGTTGCAG-3′
IGF	Forward: 5′-GCTGGTGGAAGCTCTTCAGTTC-3′
	Reverse: 5′-AGCTGACTTGGCAGGCTTGAG-3′
GAPDH	Forward: 5′-GGCTCTCTGCTCCTCCCTGTT-3′
	Reverse: 5′-GGCTCTCTGCTCCTCCCTGTT-3′

bFGF, basic fibroblast growth factor; GAPDH, glyceraldehyde 3-phosphate dehydrogenase; HGF, hepatocyte growth factor; IGF, insulin-like growth factor; MIF, macrophage migration inhibitory factor; VEGF, vascular endothelial growth factor.

### Western blot

Western blot analyses were carried out as described previously [29]. Briefly, cells and tissue samples were washed twice with ice-cold phosphate-buffered saline and ruptured with lysis buffer containing 20 mM Tris–HCl, 150 mM NaCl, 1% Triton X-100, and protease and phosphatase inhibitors. Tissue samples were further homogenized using a rotorstator homogenizer. The lysates were centrifuged for 5 minutes at 12,000 × *g*; the supernatant consisted of total cellular protein. For each sample, 20 μg total protein was resolved by SDS-PAGE and transferred onto PVDF polyvinylidene difluoride membranes. Membranes were blocked for 1 hour with 5% skim milk in Tris-buffered saline containing 0.1% Tween 20, and incubated overnight at 4°C with primary antibodies. The following day, membranes were washed, incubated for 1 hour with appropriate secondary antibodies conjugated to horseradish peroxidase, and developed using chemiluminescent substrates. The stained protein bands were visualized on BIO-RAD ChemiDoc XRS equipment (Hercules, BIO-RAD, CA, USA), and quantified and analyzed using the Quantity One software (Hercules, BIO-RAD, CA, USA).

### Immunofluorescent staining

To examine the expression of CD74 on the surface of MSCs, cells were first grown on glass coverslips, fixed with 4% paraformaldehyde for 15 minutes at room temperature, blocked with 10% bovine serum albumin and incubated with anti-CD74 primary antibody at 4°C overnight. The following day, coverslips were washed, and cells were incubated with Alexa Fluor 555-conjugated goat anti-rabbit IgG for 1 hour at 37°C. Finally, the nuclei were counterstained with 4,6-diamidino-2-phenylindole. Images were acquired using a fluorescence microscope (Leica DMI4000 B; Leica, Wetzlar, Germany).

### Statistical analysis

Data are expressed as mean ± standard deviation. Statistical significance of differences among groups was tested by one-way analysis of variance. Comparisons between two groups were performed using Student’s *t* test. *P* <0.05 was considered statistically significant.

## Results

### Downregulation of MIF expression in aged heart

**Figure 1:**
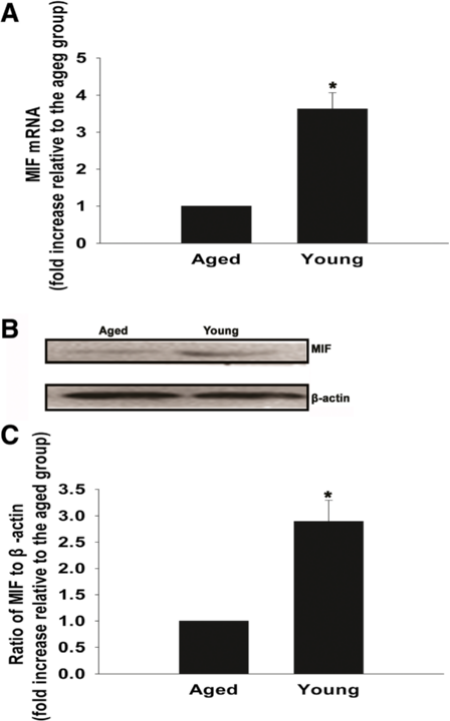
Downregulation of macrophage migration inhibitory factor expression in aged heart. **(A)** Quantitative real-time PCR analysis of macrophage migration inhibitory factor (MIF) mRNA levels in aged and young heart tissue. **(B), (C)** Western blot analysis of MIF protein levels in aged and young heart tissue. Each column represents the mean ± standard deviation from three independent experiments; *P <0.05 versus aged heart tissue.

First, we examined the expression levels of MIF in both young and aged rat hearts. We found that both mRNA and protein expression of cardiac MIF was markedly decreased in the older hearts (Figure 1), supporting our hypothesis that the decrease in MIF expression and activity is associated with ageing.

### Expression and secretion of MIF is downregulated in aged MSCs

**Figure 2:**
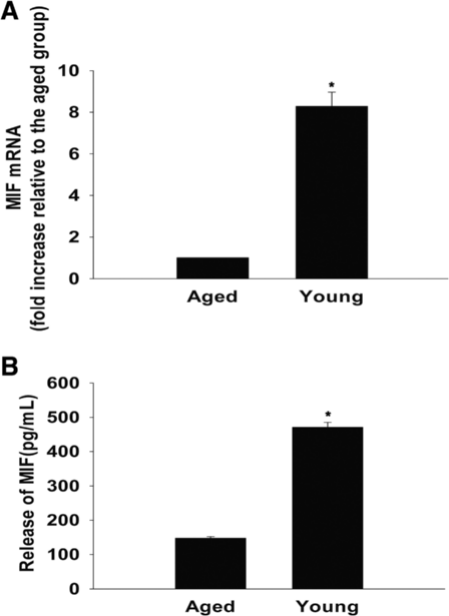
Reduced expression and release of macrophage migration inhibitory factor in aged mesenchymal stem cells. **(A)** Macrophage migration inhibitory factor (MIF) mRNA levels analyzed by quantitative real-time PCR in aged and young mesenchymal stem cells (MSCs). **(B)** Concentration of MIF in the culture medium, analyzed by enzyme-linked immunosorbent assay in cultures of aged and young MSCs. Each column represents mean ± standard deviation from three independent experiments; *P <0.05 versus aged.

First, we examined the basal level of expression of MIF mRNA in aged and young MSCs using quantitative real-time PCR. MIF transcript level in young cells was approximately eightfold higher than the older MSCs (Figure 2A). Consistent with this, we also found a twofold to threefold increase in the concentration of secreted MIF in the culture medium of young MSCs in comparison with the aged cells (Figure 2B).

### Exogenous MIF treatment restores the survival and function of aged MSCs in a concentration-dependent manner

**Figure 3:**
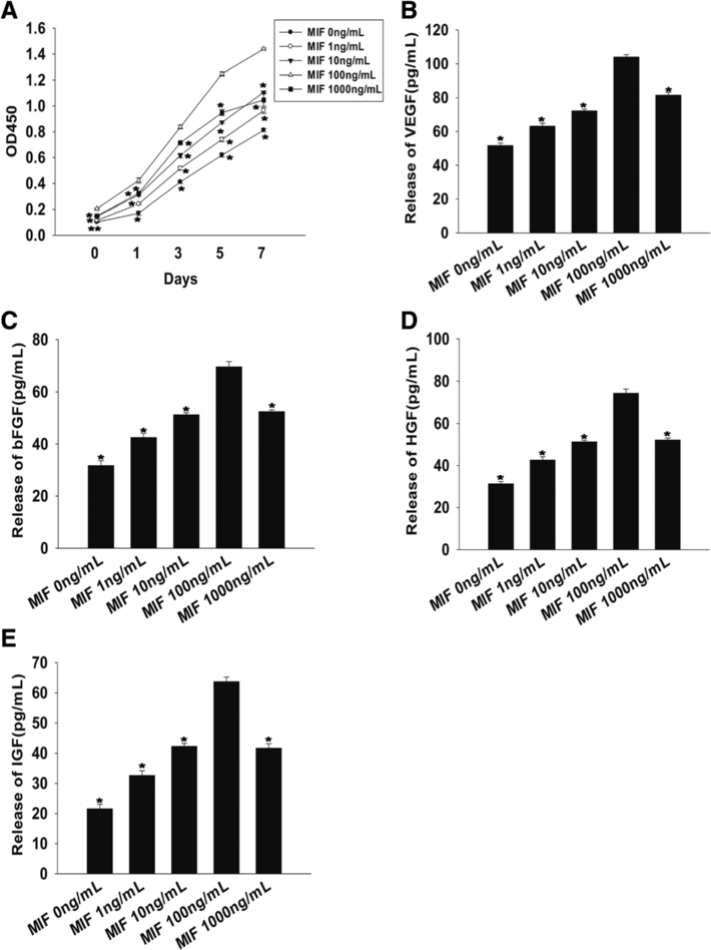
Macrophage migration inhibitory factor induces rejuvenation in a concentration-dependent manner. **(A)** Proliferation growth curves of mesenchymal stem cells incubated with macrophage migration inhibitory factor (MIF) at concentrations of 1 to 1,000 ng/ml, determined by the Cell Counting Kit-8 (HaiGene Technology, Harbin, China) assay during 1, 3, 5 and 7 days of treatment. Each data point represents mean ± standard deviation from three independent experiments; *P <0.05 versus 100 ng/ml MIF. Relative concentration of **(B)** vascular endothelial growth factor (VEGF), **(C)** basic fibroblast growth factor (bFGF), **(D)** hepatocyte growth factor (HGF) and **(E)** insulin-like growth factor (IGF) analyzed by enzyme-linked immunosorbent assay. Each column represents mean ± standard deviation from three independent experiments; *P <0.05 versus 100 ng/ml MIF.

Previous studies have shown that MIF promotes the survival and proliferation of neural stem/progenitor cells as well as B cells at an approximate concentration of 100 ng/ml [17,27]. To determine the optimal concentration for MIF function, we used a range of 1 to 1,000 ng/ml to treat aged MSCs. Next, we assayed their proliferation and found that a concentration of 100 ng/ml not only induced the maximum rate of proliferation (Figure 3A) but also induced the largest trophic effects, indicated by the highest levels of secreted VEGF, bFGF, HGF and IGF (Figure 3B,C,D,E).

**Figure 4:**
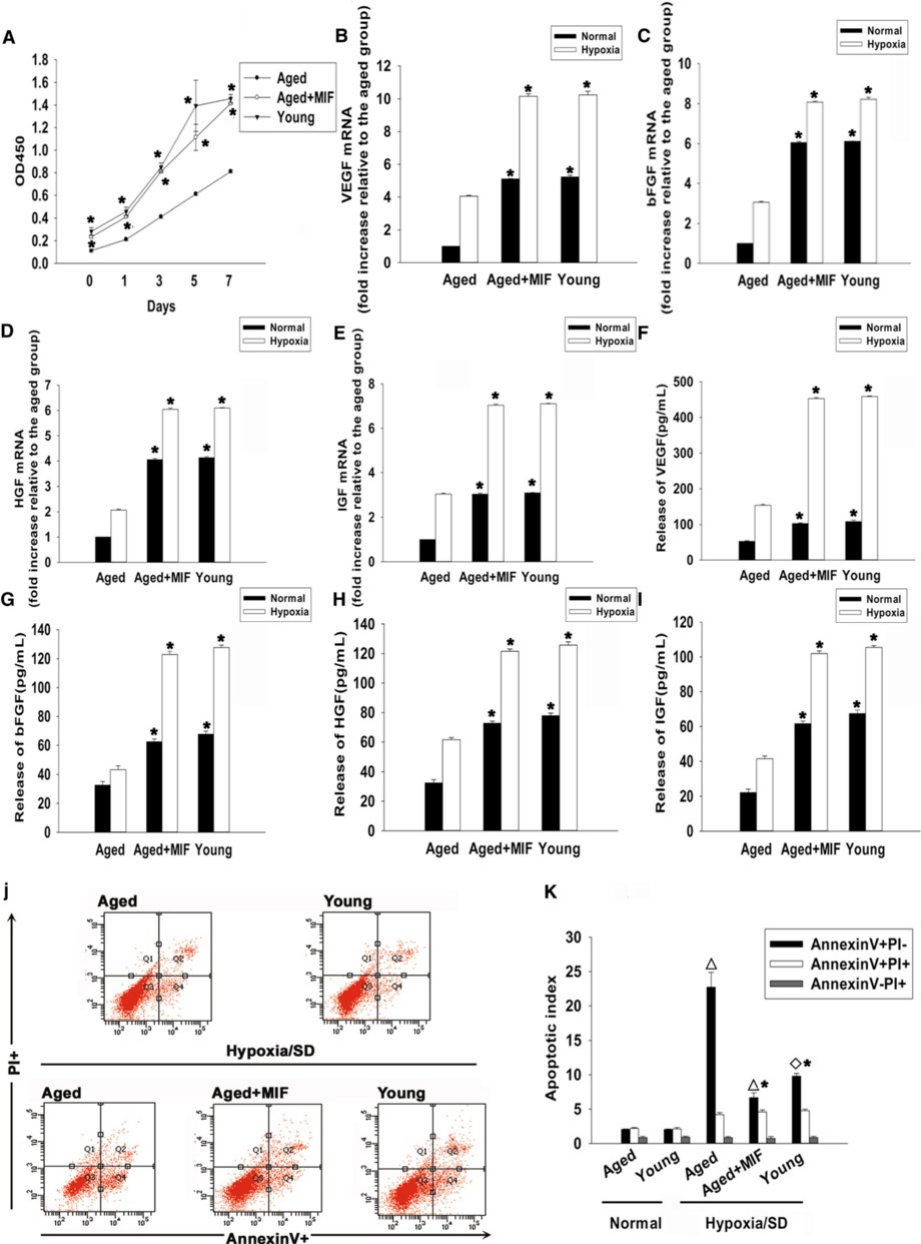
Effect of macrophage migration inhibitory factor on proliferation, paracrine signaling and survival of mesenchymal stem cells. **(A)** Proliferation growth curves of mesenchymal stem cells (MSCs), determined by the Cell Counting Kit-8 (HaiGene Technology, Harbin, China) assay, in young MSCs, aged MSCs and aged MSCs treated with macrophage migration inhibitory factor (MIF; 100 ng/ml) during 1, 3, 5 and 7 days of treatment. Each data point represents mean ± standard deviation from three independent experiments; *P <0.05 versus aged. mRNA levels of **(B)** vascular endothelial growth factor (VEGF), **(C)** basic fibroblast growth factor (bFGF), **(D)** hepatocyte growth factor (HGF) and **(E)** insulin-like growth factor (IGF) analyzed by quantitative real-time PCR in the culture of young, aged and MIF-treated aged MSCs, under normal and hypoxic conditions. Each column represents mean ± standard deviation from three independent experiments; *P <0.05 versus aged. Relative concentration of **(F)** VEGF, **(G)** bFGF, **(H)** HGF and **(I)** IGF analyzed by enzyme-linked immunosorbent assay, in the culture medium of young, aged and MIF-treated aged MSCs under normal and hypoxic conditions. Each column represents mean ± standard deviation from three independent experiments; *P <0.05 versus aged. **(J)** Representative distributions of propidium iodide (PI) and Annexin V staining from three FACScan flow cytometric analyses of apoptotic cells in normal and hypoxic conditions, in cultures of young, aged and MIF-treated (100 ng/ml added at the point of exposure to hypoxia and serum deprivation (hypoxia/SD) and maintained as such for 6 hours) MSC cultures: live (bottom left, Q-III), necrotic (top left, Q-I), early apoptotic (bottom right, Q-IV), late apoptotic (top right, Q-II). **(K)** Fold-change of apoptotic cells compared with corresponding control cells. Each column represents mean ± standard deviation from three independent experiments. *P <0.05 versus hypoxia/SD + aged, ^**△**^ *P* <0.05 versus normal + aged, ^◇^ *P* <0.05 versus normal + young.

To determine the effect of MIF exposure on the biological properties of aged MSCs, we first examined the self-renewal potential of young and old MSCs using the CCK-8 assay, and confirmed findings from previous reports that demonstrated low rates of proliferation in aged MSCs [30]. Interestingly, when we treated the aged MSCs with MIF the proliferation rate increased significantly starting from 1 day of treatment up to at least 7 days, at which point the cultures started to resemble young MSCs (Figure 4A).

MSCs secrete a variety of cytokines and growth factors that can function in both a paracrine and an autocrine manner. Such trophic effects of MSCs are known to be impaired by senescence [31]. To determine whether MIF can restore the trophic activity of MSCs isolated from aged bone marrow, we performed quantitative real-time PCR analysis of VEGF, bFGF, HGF and IGF expression. As expected, in comparison with younger bone marrow MSCs, the mRNA levels of all four factors was significantly lower in aged cells, under both normal and hypoxic conditions. Importantly, when we treated aged MSCs with MIF, this difference was abolished in both normal and hypoxic conditions (Figure 4B,C,D,E). Next, we performed enzyme-linked immunosorbent assays to quantify the levels of VEGF, bFGF, HGF and IGF in the culture media of aged MSCs with and without MIF treatment, and found an effect consistent with above results. MIF treatment significantly enhanced the secretion of all four factors under both normal and hypoxic conditions. Moreover, as seen before, the levels of secreted trophic factors in MIF-exposed aged MSC cultures were similar to the levels detected in cultures of young MSC (Figure 4F,G,H,I).

Senescence reduces the regenerative potential of MSCs, and is one of main reasons for increased susceptibility of aged MSCs to apoptotic cell death under ischemic conditions [18]. Hence, we examined the effect of MIF on the survival of aged MSCs, and found that these cells were significantly less apoptotic than MSCs which were not exposed to MIF. Interestingly however, MIF-treated aged MSCs survived better than even young MSCs not treated with MIF (Figure 4J,K). This suggests that MIF not only has a restorative function on senescent MSCs, but might also possess anti-apoptotic properties.

### Rejuvenation of aged MSCs by MIF is mediated through CD74-dependent signaling

**Figure 5:**
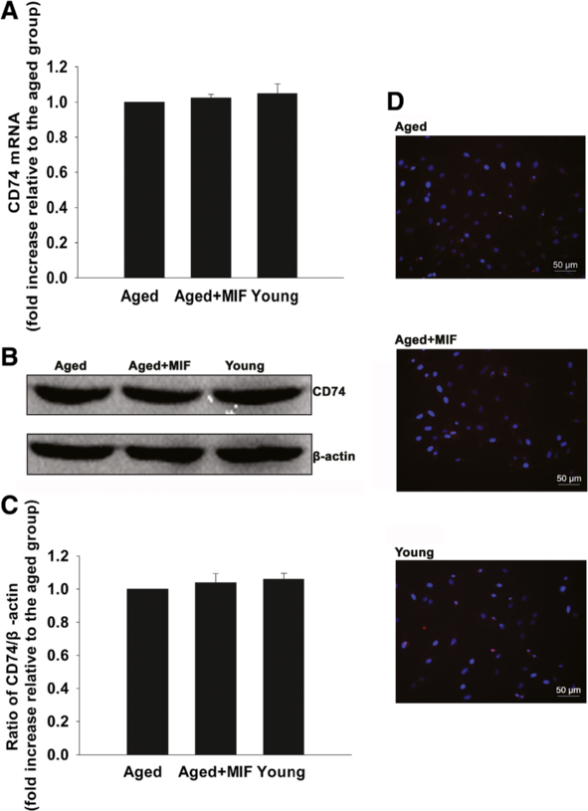
Effect of macrophage migration inhibitory factor on CD74 expression in mesenchymal stem cells. Expression of macrophage migration inhibitory factor (MIF) **(A)** mRNA analyzed by quantitative real-time PCR and **(B)** protein analyzed by western blot. Each column represents mean ± standard deviation from three independent experiments; *P* >0.05. **(C)** Densitometric quantification of MIF expression relative to internal control β-actin in young, aged and MIF-treated aged mesenchymal stem cells (MSCs). **(D)** Immunofluorescent staining of CD74 in young, aged and MIF-treated aged MSCs.

**Figure 6:**
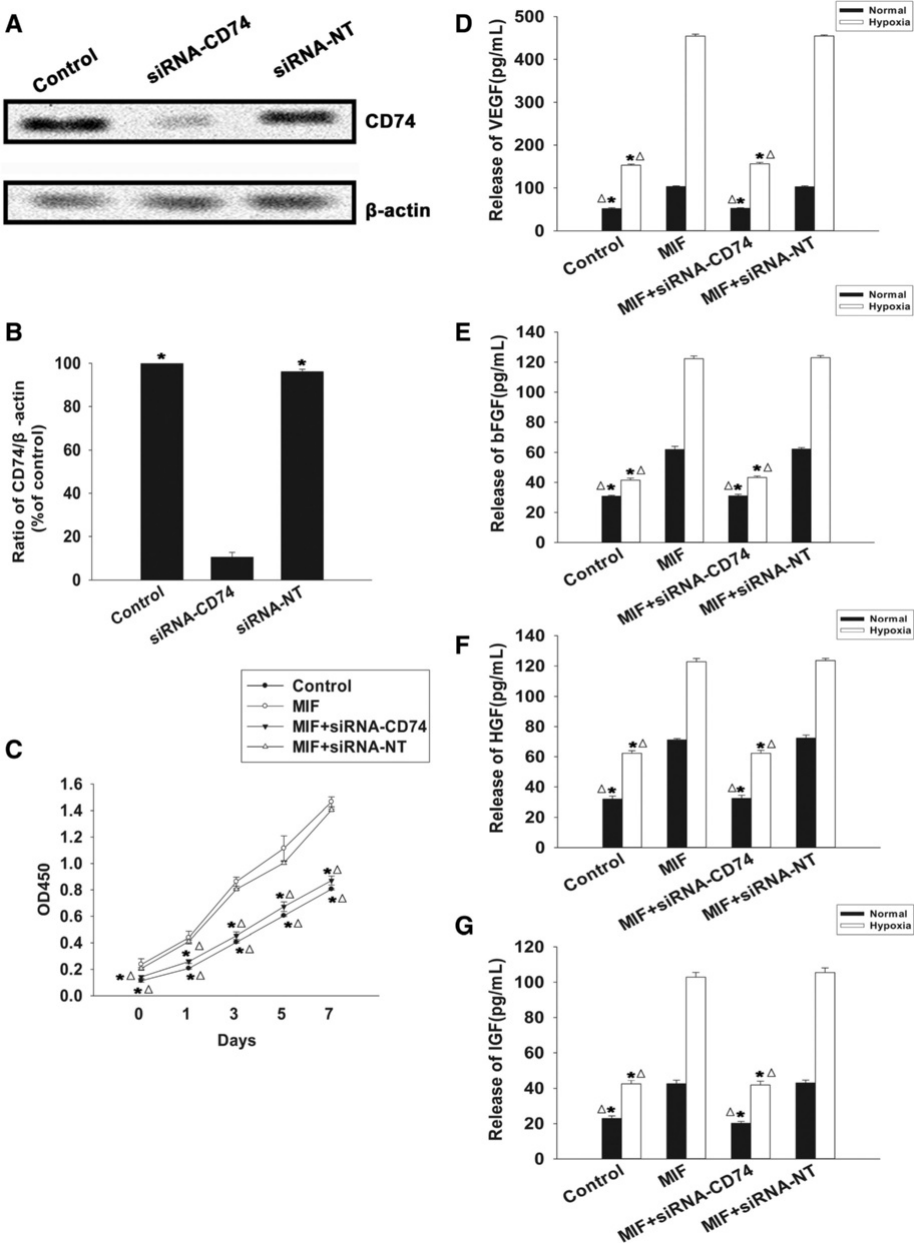
Macrophage migration inhibitory factor function is mediated through CD74. **(A)** Western blot analysis of CD74 expression in untransfected mesenchymal stem cells (MSCs), and MSCs transfected with CD74-specific small interfering RNA (siRNA) and nontarget specific control scrambled small interfering RNA (siRNA-NT). **(B)** Densitometric quantification of CD74 expression relative to internal control β-actin in all three conditions. Each column represents mean ± standard deviation from three independent experiments; *P <0.05 versus siRNA-CD74. **(C)** Proliferation growth curves (determined by the Cell Counting Kit-8 (HaiGene Technology, Harbin, China) assay) of untransfected and untreated MSCs, and macrophage migration inhibitory factor (MIF)-treated control MSCs, CD74-siRNA transfected MSCs and siRNA-NT transfected MSCs. Each data point represents mean ± standard deviation from three independent experiments; *P <0.05 versus MIF; ^△^ *P* <0.05 versus MIF + siRNA-NT. (D,E,F,G) Concentration of **(D)** vascular endothelial growth factor (VEGF), **(E)** basic fibroblast growth factor (bFGF), **(F)** hepatocyte growth factor (HGF) and **(G)** insulin-like growth factor (IGF) under normal and hypoxic conditions, in the culture medium of untransfected and untreated MSCs, and MIF-treated control MSCs, CD74-siRNA transfected MSCs and siRNA-NT transfected MSCs. Each column represents mean ± standard deviation from three independent experiments; *P <0.05 versus MIF; ^△^ *P* <0.05 versus MIF + siRNA-NT.

**Figure 7:**
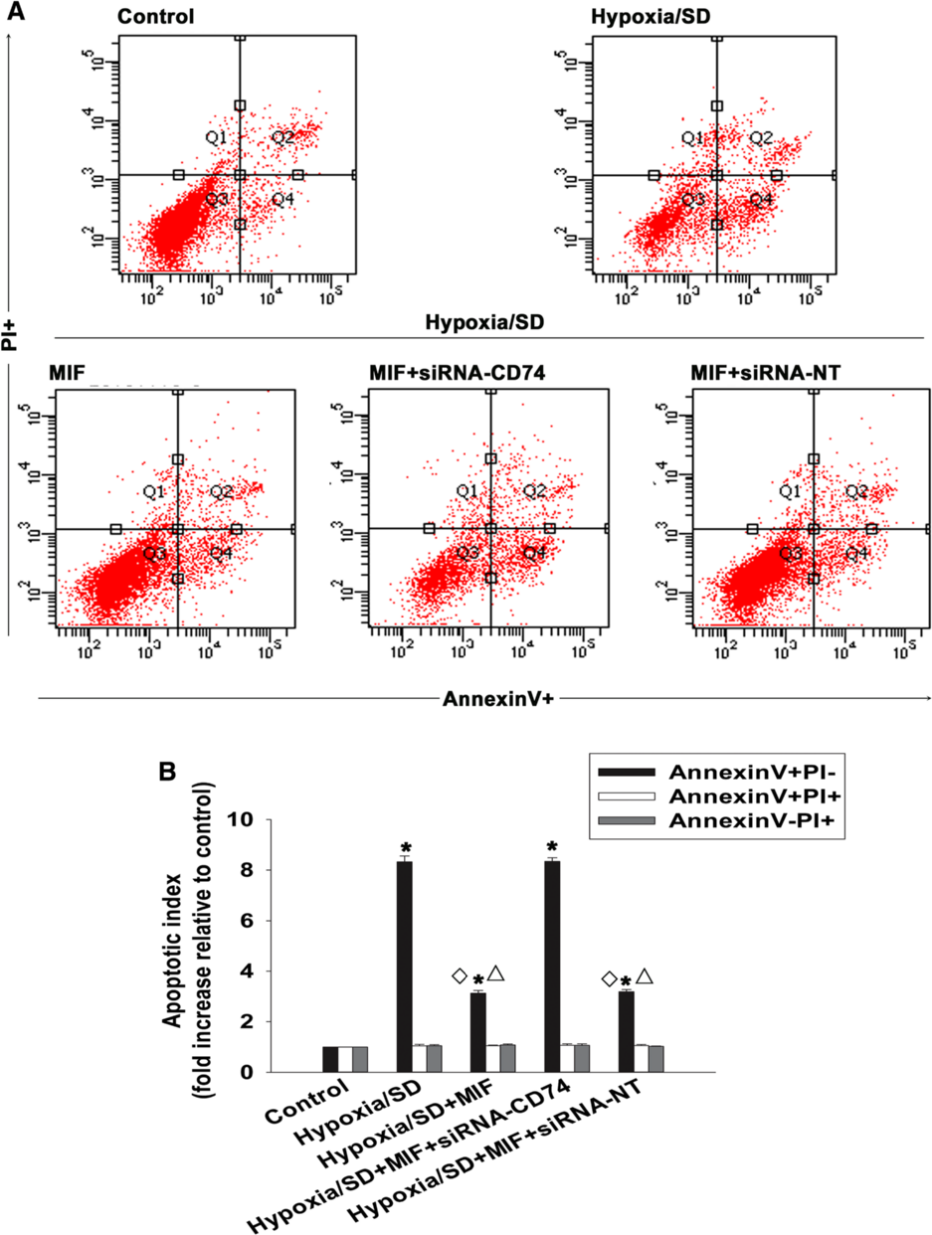
Macrophage migration inhibitory factor restores cell survival through CD74. **(A)** Representative distributions of propidium iodide (PI) and Annexin V staining from FACScan flow cytometric analyses of apoptotic cells in normal and hypoxia and serum deprivation (hypoxia/SD) (6 hours) conditions, in cultures of untransfected and untreated mesenchymal stem cells (MSCs), and macrophage migration inhibitory factor (MIF)-treated (100 ng/ml at the point of exposure to hypoxia/SD) control MSCs, CD74-small interfering RNA (siRNA) transfected MSCs and scrambled small interfering RNA (siRNA-NT) transfected MSCs. MIF was added to the incubation medium throughout the hypoxia/SD treatment period. **(B)** Fold-change of apoptotic cells in above conditions, compared with control. Each column represents mean ± standard deviation from three independent experiments; *P <0.05 versus control; ^△^ *P* <0.05 versus hypoxia/SD; ^◇^ *P* <0.05 versus hypoxia/SD + siRNA-CD74.

CD74 is largely recognized as a receptor of MIF [27,32]. To elucidate the molecular mechanism underlying the aforementioned restorative functions of MIF, we examined the effect of MIF treatment on the activity of CD74-dependent signaling. First, we assayed the expression of CD74 quantitative real-time PCR, western blot and immunofluorescence, and found no differences between aged MSCs, MIF-treated aged MSCs and young MSCs (Figure 5A,B,C,D). To further analyze the role of CD74, we knocked down its expression in aged MSCs using siRNAs, and examined subsequent rates of proliferation, paracrine signaling and survival in aged MSCs, with and without MIF treatment. As shown in Figure 6A,B, siRNA transfection significantly decreased the mRNA and protein levels of CD74, indicating efficient targeting of gene expression by the specific siRNA used in this experiment. More importantly, silencing of CD74 significantly attenuated the rejuvenative effect of MIF, as seen by the decrease in proliferation (Figure 6C), paracrine secretion of VEGF, bFGF, HGF and IGF under both normal and hypoxic conditions (Figure 6D,E,F,G), and an increase in apoptotic cell death (Figure 7A,B). In contrast, we found no differences in any of these parameters when aged MSCs were transfected with control siRNA-NT.

### **Macrophage migration inhibitory factor exposure leads to activation of the AMPK**–**FOXO3a signaling pathway**

**Figure 8:**
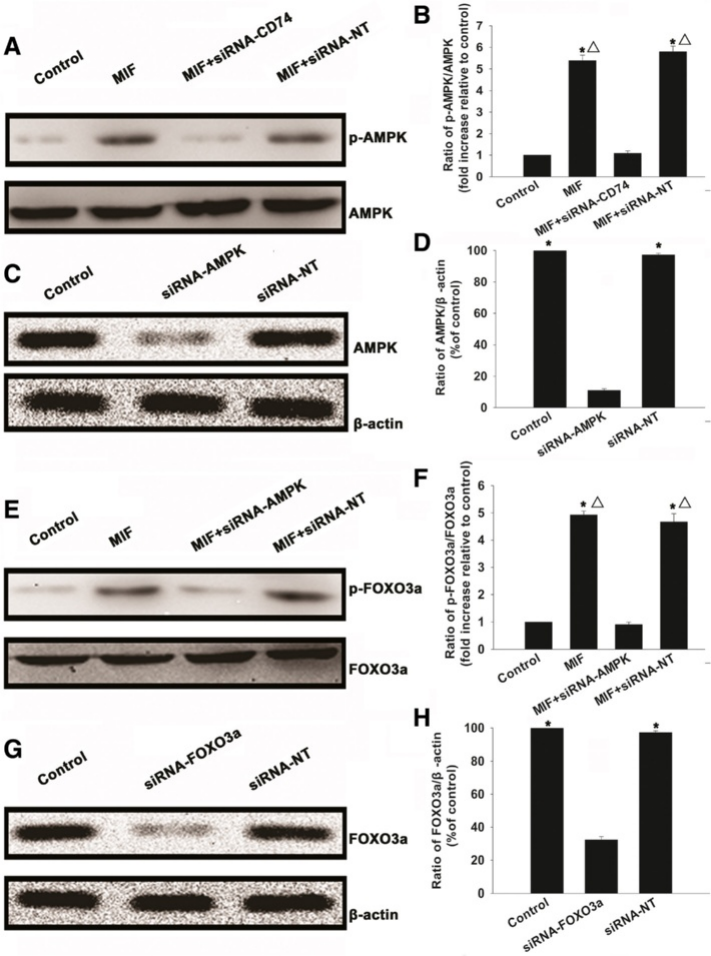
Macrophage migration inhibitory factor induces CD74-dependent activation of the AMPK-FOXO3a signaling pathway. **(A, B)** Representative images of western blots of AMP-activated protein kinase (AMPK) and phospho-AMPK in mesenchymal stem cells (MSCs) transfected with CD74-small interfering RNA (siRNA) or scrambled small interfering RNA (siRNA-NT) before pretreatment with macrophage migration inhibitory factor (MIF) (100 ng/ml) and incubated in normal conditions for the indicated time. Fold-changes were calculated normalized to AMPK. Each column represents mean ± standard deviation from three independent experiments; *P <0.05 versus control; ^△^ *P* <0.05 versus siRNA-CD74. **(C, D)** Representative images of western blots of AMPK and β-actin in MSCs transfected with siRNA against AMPK genes, and siRNA-NT. Each column represents mean ± standard deviation from three independent experiments; *P <0.05 versus siRNA-AMPK. Fold-changes were calculated normalized to β-actin. **(E, F)** Representative images of western blots of Forkhead box class O 3a (FOXO3a) and phospho-FOXO3a in MSCs transfected with siRNA-AMPK or siRNA-NT before pretreatment with MIF (100 ng/ml) and incubated under normal conditions for the indicated time. Fold-changes were calculated normalized to FOXO3a. Each column represents mean ± standard deviation from three independent experiments; *P <0.05 versus control; ^△^ *P* <0.05 versus siRNA-AMPK. **(G, H)** Representative images of western blots of FOXO3a and β-actin in MSCs transfected with siRNA against FOXO3a genes, and siRNA-NT. Fold-changes were calculated normalized to β-actin. Each column represents mean ± standard deviation from three independent experiments; *P <0.05 versus siRNA-FOXO3a.

The AMPK–FOXO3a signaling pathway is an important regulator of senescence in many cell types [33,34]. Therefore, we next investigated whether this mechanism is involved in mediating the restorative effects of MIF in MSCs. As shown in Figure 8A,B, western blot analysis revealed low levels of detectable phospho-AMPK in control cells. In contrast, MIF exposure induced a pronounced increase in the phosphorylation, and hence activation, of AMPK, which was abolished by siRNA-mediated silencing of CD74 expression. No differences were observed in cells transfected with siRNA-NT. Similarly, MIF exposure also caused a significant increase in the phosphorylation of FOXO3a in MSCs. To determine whether activation of FOXO3a is dependent on the activation AMPK, we silenced AMPK by transfecting specific siRNAs, which significantly downregulated the expression of AMPK. Silencing of AMPK expression abolished the phosphorylation of FOXO3a subsequent to MIF treatment, with no changes detected in the siRNA-NT transfected cells (Figure 8E,F).

### Rejuvenative function of MIF is mediated through CD74-dependent AMPK–FOXO3a signaling

**Figure 9:**
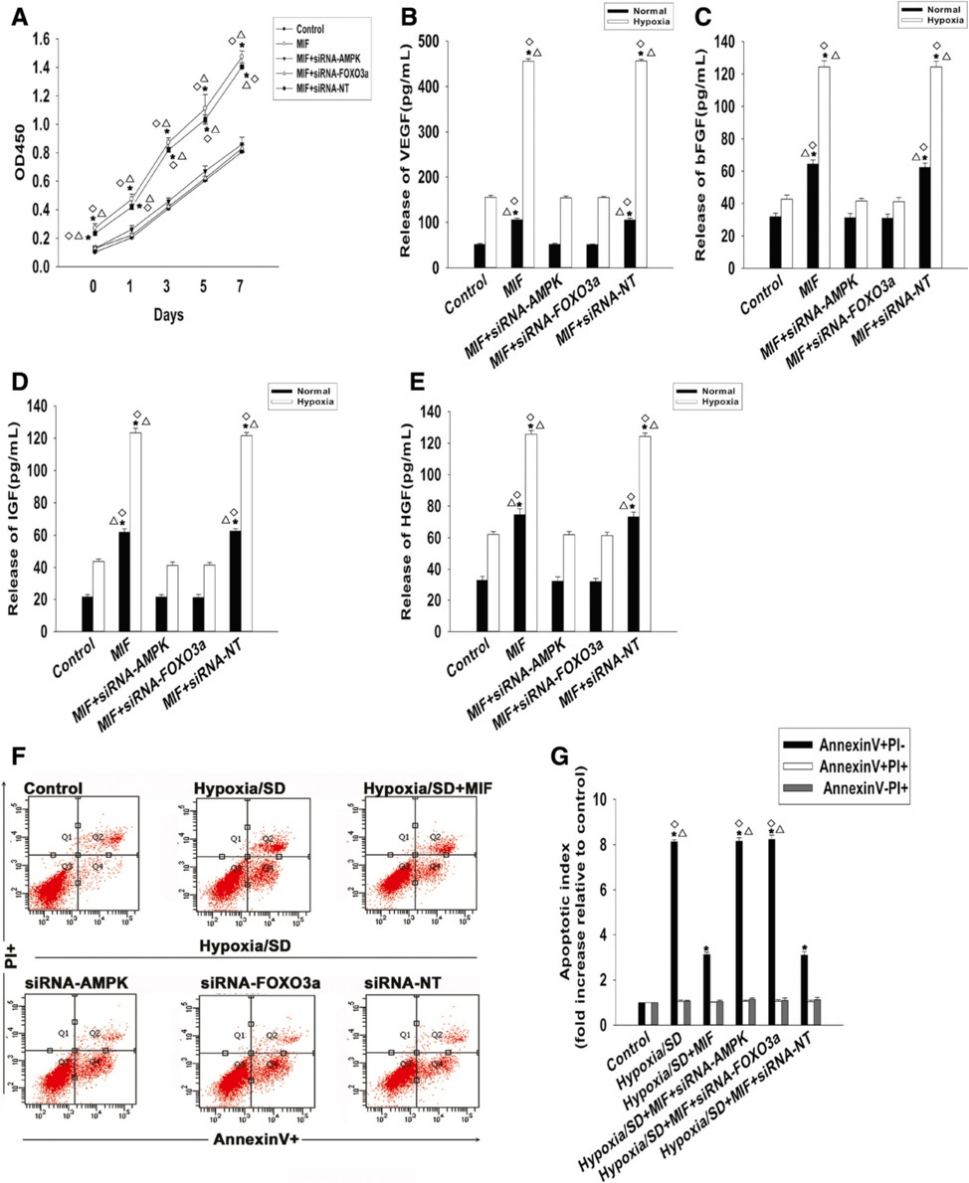
Macrophage migration inhibitory factor restores cell rejuvenation through CD74-dependent AMPK–FOXO3a signaling. **(A)** Proliferation growth curves of mesenchymal stem cells (MSCs) transfected with small interfering RNA (siRNA)-AMP-activated protein kinase (AMPK), siRNA-Forkhead box class O 3a (FOXO3a), or scrambled small interfering RNA (siRNA-NT) control, and treated with macrophage migration inhibitory factor (MIF). Each data point represents mean ± standard deviation from three independent experiments; *P <0.05 versus control; ^△^ *P* <0.05 versus MIF + siRNA-AMPK; ^◇^ *P* <0.05 versus MIF + siRNA-FOXO3a. Concentration of **(B)** vascular endothelial growth factor (VEGF), **(C)** basic fibroblast growth factor (bFGF), **(D)** hepatocyte growth factor (HGF) and **(E)** insulin-like growth factor (IGF) under normal and hypoxic conditions, in the culture medium of MSCs transfected with siRNA-AMPK, siRNA-FOXO3a or siRNA-NT control, and treated with MIF. Each column represents mean ± standard deviation from three independent experiments; *P <0.05 versus control; ^△^ *P* <0.05 versus MIF + siRNA-AMPK; ^◇^ *P* <0.05 versus MIF + siRNA-FOXO3a. **(F)** Representative distributions of propidium iodide (PI) and Annexin V staining from FACScan flow cytometric analyses of apoptotic cells in normal and hypoxia and serum deprivation (hypoxia/SD) (6 hours) conditions, in cultures of MSCs transfected with siRNA-AMPK, siRNA-FOXO3a or siRNA-NT control, and treated with MIF (100 ng/ml at the point of exposure to hypoxia/SD). MIF was added to the incubation medium throughout the hypoxia/SD treatment period. **(G)** Fold-change of apoptotic cells in the above conditions, compared with control. Each column represents mean ± standard deviation from three independent experiments; *P <0.05 versus control; ^△^ *P* <0.05 versus hypoxia/SD + MIF; ^◇^ *P* <0.05 versus hypoxia/SD + siRNA-NT.

The data above suggest that AMPK–FOXO3a signaling is likely to be involved in the mechanism of action of MIF in aged MSCs. In order to elucidate this hypothesis, we first silenced AMPK and FOXO3a expression using siRNAs. As shown in Figure 8, siRNA-AMPK and siRNA-FOXO3a significantly downregulated the expression of AMPK and FOXO3a, respectively (Figure 8C,D,G,H). Next, we analyzed the effect of the knockdown on the proliferation, paracrine signaling and survival of aged MSCs under a hypoxia/SD condition. As shown in Figure 9A, knockdown of both AMPK and FOXO3a decreased the extent of proliferation starting from 1 day of treatment up to at least 7 days. Transfection with siRNA-NT did not lead to any changes. The knockdown also impaired the secretion of VEGF, bFGF, HGF and IGF, under both normal and hypoxic conditions (Figure 9B,C,D,E), and abolished the anti-apoptotic effects of MIF. The control siRNA-NT did not cause any alterations in cell survival (Figure 9F,G).

## Discussion

Autologous MSCs offer a great advantage when transplanted into ischemic or infarcted heart to regenerate and repopulate the injured myocardium and restore heart function. They are immunologically safe and easy to prepare from adult patients [35]. However, like other stem cells, MSCs are susceptible to age-related changes, including increased rates of apoptosis and senescence, and decreased rates of proliferation and paracrine signaling [36-38], which reduce their ability to contribute to endogenous injury repair processes [39]. Numerous approaches have been attempted to overcome these limitations, and some have led to dramatic improvements in cardiac function, specifically in a rodent model of acute myocardial infarction [30]. However, despite these successes, researchers continue to explore ways to make the regenerative process easier to achieve and more effective in restoring biological function.

Results of the current study suggest that MIF treatment can effectively rejuvenate MSCs isolated from age-induced senescent rats. We further show that this function is mediated through activation of the CD74-dependent AMPK–FOXO3a signaling pathway, leading to increased proliferation and paracrine signaling activity and decreased hypoxia/SD-induced apoptosis. Our data strongly suggest that MIF is a promising candidate for a rejuvenating agent for application in cell transplantation therapy in age-induced senescent patients.

Aging is an important risk factor for cardiovascular diseases. Furthermore, with the onset of such conditions, which often occurs secondary to atherosclerotic plaque-induced narrowing of blood vessels, the function of both resident and circulating stem and progenitor cells is diminished [40,41]. The cumulative effect of these disease-related and age-related deficits may contribute to a severe decrease in the proliferation, paracrine signaling and survival of stem cells [30]. Here, we show that aged MSCs can regain their biological properties following exposure to MIF, in most cases, to the extent that they start to resemble young MSCs. Specifically, they display increased proliferation rates, paracrine function and resistance to apoptosis. Our data corroborate previous findings that MIF exerts an anti-apoptotic effect in cardiomyocytes exposed to an ischemic environment [16,42]. Here, we show that aged MSCs, when treated with MIF, show a lower degree of early apoptosis than young untreated MSCs. Furthermore, researchers have shown that circulating MIF levels are increased during myocardial infarction but diminished during aging, suggesting that MIF-mediated signaling and its protective effects are active during cardiac ischemia but impaired by senescence [43].

MIF is a proinflammatory cytokine, originally identified to play an important role in chronic inflammatory diseases [44]. MIF also contributes to cell survival and proliferation, and prevents cellular senescence [15,17]. Mechanisms underlying MIF-dependent biological functions are still being investigated, but have been shown to involve activity of the AMPK, mitogen-activated protein kinase/extracellular signal-regulated kinase and phosphoinositide 3-kinase/Akt signaling pathways. There is large evidence that these mechanisms are important for cellular proliferation, survival and senescence [11,18,45]. Endogenous MIF seems to exert a protective effect to modulate the cellular energy state leading to increased ATP production and limited energy consumption, especially in conditions such as glucose deprivation, ischemia, hypoxia, oxidative stress and senescence. With regard to senescence, studies have shown that MIF expression is reduced in aged hearts [15]. Previous studies have shown that cardiomyocytes in mice deficient in MIF (MIF^−/−^) exhibit contractile defects in response to starvation [46], and undergo increased apoptosis during ischemia/reperfusion *in vivo* [47]. In addition, mice deficient in either MIF (MIF^−/−^) or the MIF receptor CD74 (CD74^−/−^) activate the expression of markers of senescence pathways p53/21 and p16, and develop spontaneous emphysema by 6 months of age [48]. We corroborated these findings in our study, and showed significantly decreased expression of MIF in aged heart tissue, in comparison with younger hearts. Interestingly, we also found that despite the reduced basal level of expression, aged MSCs can also secrete MIF. In contrast, younger MSCs express MIF at higher levels. Moreover, MSCs also express CD74, suggesting that the MIF released by these cells might have autocrine function. Hence, strategies that can facilitate regaining of endogenous MIF level and activity might provide an additive effect while using MSCs to treat ischemic heart diseases, especially in aged patients.

CD74 is a well known receptor of MIF, shown to activate downstream signaling through a membrane receptor complex [11,32,49]. MIF binds to CD74 through its N-terminal region, which is also the site of its intrinsic tautomerase activity, considered to be vestigial and nonphysiological [32]. Consistent with previous reports, we found no difference in CD74 expression between aged and young MSCs. Interestingly, although treatment with MIF did not influence CD74 expression in MSCs, siRNA-mediated knockdown of CD74 in the latter significantly diminished the rejuvenating effect of MIF. This strongly suggests that the regenerative functions of MIF are mediated through CD74-dependent signaling, consistent with studies that showed improved survival and proliferation of neural stem/progenitor cells and B cells in response to MIF exposure through activation of a CD74-dependent pathway [17,27].

AMPK is a kinase involved in stress signaling, and is a key regulator of pathways that control energy generation and consumption [50,51]. Cellular aging might be the consequence of either the exhaustion of a metabolite or a regulatory factor, or the unavoidable accumulation of damage, including proteotoxicity, and oxidative, metabolic and DNA damage [52-54]. In higher organisms, AMPK plays a critical role in metabolism and stress-associated cellular processes [55]. Studies have suggested that AMPK signaling is involved in regulating efficient energy consumption, by mediating metabolic homeostasis, enhanced stress resistance and qualified cellular housekeeping, which are the hallmarks of improved health and extended lifespan [56]. There is emerging evidence that the responsiveness of AMPK signaling clearly declines with age [57]. This loss of sensitivity to cellular stress in turn impairs metabolic regulation, increases oxidative stress and reduces autophagic clearance [56]. In the present study we show that MIF exposure leads to phosphorylation and activation of AMPK, consistent with other studies in which several upstream kinases, including serine/threonine kinase 11 (LKB1) [58], Ca^2+^/calmodulin-dependent protein kinase kinase β (CaMKKβ) [59] and transforming growth factor-beta-activated kinase 1 (TAK1) [60], were shown to activate AMPK by phosphorylating the catalytic alpha subunit at Thr^172^. This in turn enabled effective regulation of energy metabolism and cellular homeostasis. We further show that activation of AMPK is mediated through a CD74-dependent pathway, since silencing of CD74 expression in MSCs led to impaired phosphorylation of AMPK. In addition, siRNA-mediated silencing of AMPK led to a significant decline in the restoration of cellular function in aged MSCs in response to MIF. This suggests that AMPK-mediated signaling is essential for the rejuvenating activity of MIF.

FOXO3a is a member of the mammalian FoxO family of Forkhead transcription factors, and is involved in the regulation of several important cellular functions, including apoptosis, cell cycle, stress resistance, glucose and lipid metabolism and inflammation [61,62]. FOXO3a is also a key player in the regulation of longevity [22], and has been closely associated with cellular senescence [63]. FOXO3a can inhibit nuclear factor-κB signaling through the activation of inhibitory κB genes, IκBβ and IκBε, thus preventing age-related inflammatory responses [64]. Furthermore, AMPK/FOXO3a signaling is known to inhibit the activity of p38α kinase, an inducer of cellular senescence [65]. Studies have demonstrated in mouse fibroblasts that AMPK can directly phosphorylate FOXO3a at six threonine/serine residues within the transactivation domain. They also showed that the AMPK–FOXO axis is involved in regulating lifespan and its extension [66,67]. In the current study, we show in MSCs that FOXO3a is phosphorylated at Thr^32^ in response to MIF exposure, and that this could be abolished by silencing AMPK expression. Furthermore, silencing of FOXO3a diminished MIF-induced rejuvenation of aged MSCs, suggesting that the restorative function of MIF in MSCs is mediated through the AMPK–FOXO3a signaling pathway.

In the present study, we demonstrate that exogenous supply of MIF can effectively rejuvenate the function of senescent MSCs. Using both *in vitro* and *in vivo* assays we show that senescence decreased the expression of MIF, consistent with previous findings that MIF expression is impaired under certain stress conditions. From a clinical perspective, the protective effect of exogenous MIF during ischemic heart diseases may not only be due to energy modulation, protection of cardiomyocytes and regulation of cardioprotective proteins, but also due to their rejuvenative effect on circulating stem cells. The above findings suggest that pharmacological interventions which restore MIF and related signaling pathways in the senescent heart might be useful in reducing cardiac damage caused by ischemic injury in older individuals.

## Conclusions

Our study shows that pretreatment with MIF can rejuvenate MSCs derived from the bone marrow of aged donors. Specifically, MIF can positively influence the rate of proliferation and paracrine signaling and alleviate hypoxia/SD-induced apoptosis in senescent MSCs. The demonstration that MSCs can be manipulated to cause a delay in senescence and enhance their regenerative properties has important therapeutic implications for vascular disorders. Pretreatment of MSCs with MIF might be extremely useful in cell transplantation-based repair and regeneration of peripheral vasculature and its coronary counterpart.

## Abbreviations

AMPK: AMP-activated protein kinase
bFGF: basic fibroblast growth factor
CCK-8: Cell Counting Kit-8
Ct: threshold number of cycles
FITC: fluorescein isothiocyanate
FOXO3a: Forkhead box class O 3a
HGF: hepatocyte growth factor
hypoxia/SD: hypoxia and serum deprivation
IGF: insulin-like growth factor
MIF: macrophage migration inhibitory factor
MSC: mesenchymal stem cell
siRNA: small interfering RNA
siRNA-NT: scrambled small interfering RNA
VEGF: vascular endothelial growth factor
